# Consequences of the Combined α-tocopherol, Ascorbic Acid and α-lipoic Acid on the Glutathione, Cholesterol and Fatty Acid Composition in Muscle and Liver of Diabetic Rats

**Published:** 2013-02

**Authors:** Okkes YILMAZ, Yasemin ERSAN, Ayse Dilek OZSAHIN, Ali Ihsan OZTURK, Yusuf OZKAN

**Affiliations:** 1 Department of Biology, Faculty of Science, Firat University, 23119-Elazig, Turkey; 2 Department of Biology, Faculty of Science, Bitlis Eren University, 13000-Bitlis, Turkey; 3 Department of Chemistry, Faculty of Science, Harran University, 63100-S.Urfa, Turkey; 4 Department of Internal Medicine, School of Medicine, Firat University, 23119-Elazig, Turkey

**Keywords:** Cholesterol, Diabetes, Fatty acids, Glutathione, Liver, Muscle

## Abstract

***Objective(s):*** Our objective was to evaluate the effects of a triple antioxidant combination [α-tocopherol (AT), ascorbic acid (AA) and α-lipoic acid (LA); AT+AA+LA] on the cholesterol and glutathione levels, and the fatty acid composition of liver and muscle tissues in diabetic rats.

***Materials and Methods:*** Forty-three Wistar rats were randomly divided into five groups. The first group was used as a control. The second, third and fourth groups received STZ (45 mg/kg) in citrate buffer. The fourth and fifth groups were injected with intraperitoneal (IP) 50 mg/kg DL-AT and 50 mg /kg DL-LA four times per week and received water-soluble vitamin C (50 mg/kg) in their drinking water for a period of six weeks.

***Results:*** Liver cholesterol levels in the AT+AA+LA group were lower than the control (P<0.05). Glutathione level was lower in D-2 (P<0.05) and were higher in D+AT+AA+LA and AT+AA+LA groups than the control groups (P≤ 0.05). The muscle cholesterol levels in the D-1 and D+AT+AA+LA groups were higher than the control group (P≤ 0.05). The levels of oleic acid were higher in the D-1 group and lower in the D-2 group (P<0.001). The arachidonic acid level in the D-1 and D-2 groups were lower (P<0.05), and higher in the D+AT+AA+LA group.

***Conclusion:*** Our results revealed that glutathione levels and the Stearoyl CoA Desaturase enzyme products in liver tissues of diabetic and non-diabetic rats were increased by triple antioxidant mixture.

## Introduction

Diabetes mellitus is a common degenerative disease as one of the leading global causes of morbidity and mortality (1). Increased oxidative stress and impaired antioxidant defense mechanisms are important factors in the pathogenesis and progression of diabetes (2). Clinically, diabetes is a heterogeneous disease, with a common phenotype of impaired glucose tolerance; depending on the basis of the management required to control glucose homeostasis, it can be divided into type 1 and type 2 diabetes (1, 3, 4). In the long term, diabetes leads to complications in numerous organs (5).

Evidence from experimental animals has shown the effect of diabetes on fatty acid composition liver and muscle tissues (6). Since muscle tissue lacks the capability of *de novo* synthesis of fatty acid (FA), myocytes rely on the supply of FA from extracellular sources to cover their need of these substrates. Fatty acids are supplied to cardiac and skeletal muscle either bound to plasma albumin (non-esterified fatty acids) or in the form of triacylglycerols in the core of circulating lipoproteins (7). Furthermore, changes in the muscle content of FA most likely indicate profound changes in muscle metabolism in general and in FA handling in particular. Since the metabolic fate of FA in cardiac and skeletal muscle may depend on chain length and degree of unsaturation (8), it is important to analyze the relative composition of the FA pool in these tissues. FAs are not only substantially contribute to muscle oxidative energy conversion, but also serve as substrates for biologically active compounds, such as eicosanoids, and membrane phospholipid synthesis, and act as ligands for protein factors involved in signaling transduction and gene expression. Borkman *et al* (9) stated that skeletal muscle is a main site of insulin action, and insulin sensitivity may be related to the fatty-acid composition of the phospholipids within the muscle membranes. Declined insulin sensitivity is associated with decreased concentrations of polyunsaturated fatty acids in skeletal-muscle phospholipids, raising the possibility that changes in the fatty-acid composition of muscles modulates the action of insulin. Lipid metabolism in the liver is very complex procedure and involves, among many other functions, the synthesis and secretion of very low density lipoproteins, ketone bodies and a high rate of fatty acid oxidation, which provides most of the energy that the organ requires in order accomplishing its many functions in the organism (10). Poisson and Cunnane (11) suggested that metabolism of long-chain fatty acids is impaired in both fasting and diabetes. The decrease in insulin activity simultaneously reduces the activity of the Δ-9, Δ-6, and Δ-5 desaturases with respect to fatty acids either synthesized by the animal (palmitic and stearic acids) or dietary essential (linoleic and alpha-linolenic) acids. Mimouni and Poisson (12) reported that Δ-9 desaturase activity was more depressed than the desaturase activities of either Δ-6 of Δ-5, and that insulin treatment with 10 IU/kg body weight twice a day for 2 days was able to restore the Δ- 9, 6 and 5 desaturase activities to control levels during the hypoglycemicstage . Douillet and Ciavatti (13) found that diabetes induced a decrease of monounsaturated fatty acids and particularly palmitoleic acid (16:1 n-9) in liver, aorta and plasma. They suggested that AT partially corrected Δ-9 desaturase activity and consequently enhanced monounsaturated acids in all the tissues. In addition, vitamin E treatment was normalized Δ-6 desaturase activity in the liver, increased eicosatrienoic acid (20:3 n-6) and arachidonic acid (20:4 n-6, AA) levels in the aorta. Shin et al (6) revealed that the effect of insulin on the fatty acid composition of the liver and the erythrocytes in using STZ-induced diabetic rats. They found that the activities of Δ-6 desaturase in diabetic rats were 68% of those of controls and increased to 119% of controls following insulin treatment. It has been suggested that insulin restores the fatty acid composition earlier in the liver microsome in STZ-induced diabetic rats. 

Lipoic acid plays an important role in energy metabolism. It is involved in different multienzyme complexes such as pyruvate dehydrogenase, α-ketoglutarate dehydrogenase, branched-chain α-keto acid dehydrogenase, and glycine decarboxylase complex (14, 15). Jacop *et al* (16) have indicated that parenteral administration of the antioxidant α-LA significantly enhances the capacity of the insulin-stimulated glucose transport system and of both oxidative and nonoxidative pathways of glucose metabolism in the skeletal muscle of insulin-resistant rats. Alpha tocopherol is the major chain-breaking antioxidant that protects membranes from lipid peroxidation. However, AA and reduced glutathione (GSH) will act as tocopheroxyl radical scavengers only when there is efficient removal of their oxidised forms. GSH and ascorbat circumvent lipid peroxidation via the reduction of tocopheroxyl radical to tocopherol (17). It has been reported that antioxidants such as glutathione, AT, AA, and antioxidant enzymes superoxide dismutase, catalase, glutathione reductase, and glutathione peroxidases exert synergistic actions in scavenging free radicals (18). Evidence from *in vitro* studies has suggested that antioxidant vitamins can prevent lipid peroxidation and that AA and AT have synergistic effects. However, *in vivo* evidence in support of these hypotheses is sparse (19). 

Much evidence has indicated oxidative stress as a major source of insulin resistance, whereas contemporary dietary recommendations often lack reliable information indicating the benefits of a considerable increase of antioxidants (AT, AA, LA) in the diet of subjects belonging to the high-risk group for the development of insulin resistance (20). The aim of this study was to examine the effects of triple combination of AT+AA+LA on the total cholesterol, reduced GSH levels, and fatty acid composition (products of SCD and Δ6 and Δ5 desaturase enzymes) in the liver and muscle tissues of diabetic rats. 

## Materials and Methods

*Animals, diets, experimental design*

A total 43 Wistar rats (200–250 g; 10 weaks old) were used. The animals were housed in cages in an air-conditioned room with a 12 hour light/dark cycle where they had *ad libitum *access to rat chow and water. Animals were randomly divided into five groups. The first group was used as a control (n = 6), the second group D-1 (n = 10), the third group D-2 (n = 10), the fourth group D+AT+AA+LA (n = 10), the fifth group AT+AA+LA (n =7). Rats in D-1, D-2, and D+AT+AA+LA groups were made diabetic via a single intraperitoneal injection (IP) of 45 mg/kg STZ in citrate buffer (pH = 4.5). Control group were injected IP with the buffer alone. Two days following administration of STZ, the tail vein blood glucose level was measured in all the animals. Those with blood glucose level of 250 mg/dl and above were considered diabetics. Following formation of diabetic, rats in D-1 and D+AT+AA+LA groups received daily subcutaneous injections of 8 IU/kg insulin; rats in the D-2 group were not injected. Rats in the D+AT+AA+LA group were injected intraperitoneal (IP) 50 mg / kg DL-AT and 50 mg/kg DL-LA four times per week and received water-soluble vitamin C (50 mg/kg) in their drinking water. These treatments were continued for six weeks, after which each rat was anesthetized with ether, liver and muscle tissue samples were collected and stored at –85^O^C prior to biochemical analyses.

*Laboratory analyses*

Lipids were extracted with chloroform-methanol (2:1, v/v) using the method of Folch *et al* (21) as described previously (22). Liver and muscle tissues were homogenized with the mixture of chloroform/methanol in homogenizator. Aliquots were taken and the total cholesterol quantified by high performance liquid chromatography (23). Aliquots of each extract were saponified and the fatty acids esterified with 2% H_2_SO_4 _in methanol (22) and the fatty acid composition determined by gas chromatography.

*Cholesterol analysis*

For the cholesterol analysis, fully automatic HPLC (Shimadzu, Kyoto Japan) had been used. A Supelcosil LC 18TMDB column (250 x 4.6 mm, 5 µm, Sigma USA) was used as the HPLC column. The mobile phase used was acetonitrile – isopropyl alcohol (70:30 v/v) at a flow rate of 1 ml/ min (23). Detection was performed by UV at 202 nm and 40°C column oven (24). For sample preparation, one ml of the hexane extract obtained by direct saponification was dried under nitrogen flow and diluted with a 500 µl of the mobile phase. The mixture was injected via auto sampler to the HPLC instrument. Quantification was carried out by external standardization using *Class VP software*. The results were expressed as µmol/g tissue weight.

*Determination of GSH in Liver and muscle tissues*

For the GSH analysis, a Discovery RP-Amide C16 column (150 x 4.6 mm, 5 µm) was used and 50 mM NaClO4 0.1 % H_3_PO_4_ was used as the mobile phase with a flow rate of 1 ml/min (25). Detection was performed at 215 nm by UV-Visible detector and 40 C column oven. 0.2 g liver and muscle tissue samples were homogenized with the mixture of 2 ml 10 mM ETDA and 50 mM NaClO4. Following deproteinization with 5 % metaphosphoric acid (26), samples were centrifuged at 4°C and 12,000 x g for 10 min. The supernatants were injected via an autosampler to the HPLC. Quantification was carried out by external standardization using *Class VP software*. The results were expressed as µmol/g tissue weight 

*Fatty acid methyl ester analysis*

Fatty acids in lipid extracts were converted to methyl esters by using 2% sulfuric acid (v/v) in methanol (22). Fatty acid methyl ester forms were extracted with n-hexane. Gas Chromatography (GC) analysis was employed from Shimadzu (Kyota, Japan). Methyl ester mixtures were separated by a capiller column, Permabond (Machery-Nagel, Germany). Column temperature was programmed between 130 – 220^O^C, 5^O^C/min and the final temperature was held for 15 min. Injector and FID temperatures were 240 and 280^O^C, respectively. Individual methyl esters were identified by comparison with authentic external standard mixtures analyzed under the same conditions. *Class GC 10* software version 2.01 was used to process the data. The results were expressed as percent tissue weight.

*Statistical analysis*

The experimental results were reported as mean ± SE. Statistical analysis was performed using SPSS. Analysis of variance (ANOVA) and Least Significant Difference (LSD) test were used to compare the experimental groups with the controls. 

## Results

*Total lipid, cholesterol and reduced glutathione (GSH) levels*

The effects of triple antioxidant combination on the cholesterol and GSH levels of liver are shown in Table 1. Cholesterol level did not differ between the D+AT+AA+LA and control groups (*P>*0.05), however, the cholesterol levels in D-1, D-2, and AT+AA+LA were lower than the control group (respectively, *P<* 0.05, *P<* 0.01). While the GSH level did not differ between the control and D-1 groups, its level decreased in the D-2 group (*P<*0.05). However, the GSH level increased in D+AT+AA+LA and AT+AA+LA groups (*P<*0.05, *P<*0.01). 

**Table 1:** The levels of total lipid, cholesterol and GSH in liver tissue

Groups	Total cholesterol µmol / g tissue	Reduced glutathione (GSH) µmol / g tissue
Control	9.00 ±0.49 a	0.95 ± 0.12 a
D-1	8.35 ± 0.56 a	1.06 ± 0.06 a
D-2	5.77 ±0.50 b	0.65 ± 0.05 b
D+AT+AA+LA	8.69 ±0.56 a	1.43 ± 0.28 b
AT+AA+LA	5.59 ±0.51 c	1.86 ± 0.09 c

Muscle cholesterol level was increased in D-1, D+AT+AA+LA and AT+AA+LA groups (*P<*0.05). However, the cholesterol level decreased in the D-2 group (*P<*0.01). Although GSH level did not differ between the control, D-1 and D+AT+AA+LA groups, its level decreased in the D-2 group, but its level in the AT+AA+LA group was higher than the other groups (Table 2). Fatty acid composition

**Table 2:** The levels of total lipid, cholesterol and GSH in muscle tissue

Groups	Total cholesterol µmol / g tissue	Reduced glutathione (GSH) µmol / g tissue
Control	4.29 ± 0.80 a	0.93 ± 0.14 a
D-1	6.80 ± 0.75 b	0.89 ± 0.16 a
D-2	2.82 ± 0.45 c	0.59 ± 0.11 b
D+AT+AA+LA	7.35 ± 0.90b	0.97 ± 0.32 a
AT+AA+LA	6.72 ± 0.75 b	1.24 ± 0.18 b

Values are the mean ± SE. ANOVA; one-way ANOVA P value:

a: P>0.05, b: P<0.05, c: P<0.01, d: P<0.001 using the post hoc Fischer’s P LSD test.

D-1: During the of diabetes was injected the insulin (8 IU/kg)

D-2: During the of diabetes was not injected insulin

AT+AA+LA = combination of a-TOC, ASA and a-LA

D+AT+AA+LA= Diabetes+ combination of a-TOC, ASA and a-LA

**Table 3:** The fatty acid composition in liver tissues of diabetic rats

Fatty acids	Control	D-1	D-2	D+ AT+AA+LA	AT+AA+LA
14:0	0.81±0.05	0.24 ±0.05	0.18±0.05	0.48±0.05	0.83±0.21
16:0	16.89±0.30	20.95±1.07d	22.22±0.99 d	22.23±1.05d	18.08±0.77b
18:0	24.45±1.23	18.38±0.88d	19.75±1.08 d	16.45±0.19d	21.19±0.78b
S Saturated	41.55±0.93	39.57±1.00b	38.00±1.30b	39.16±1.20b	40.10±1.65a
16:1, n 9	1.04±0.20a	0.98±0.35a	0.97±0.43a	2.50±0.59d	1.53±0.33 b
18:1, n 9	10.08±1.17a	10.22±0.14a	7.11±1.40b	15.45±1.24c	9.76±0.51d
18:1, n 7	2.11±0.35a	2.62±0.15a	3.16±0.39b	2.65±0.39b	2.69±0.12a
S MUFA	13.23±1.23a	13.82±0.92a	11.24±1.16a	20.60±0.82d	13.98±1.20a
18:2	14.39±0.80	17.32±0.47 c	19.74±1.79 d	16.37±0.75 b	18.01±0.42c
20:4	23.50±0.70 a	22.82±2.33 a	14.31±1.03 d	17.97±0.18 c	19.81±0.54c
22:6	6.76±0.64 a	6.47±0.36 a	12.74±1.53 b	5.90±0.42 a	8.12±0.11a
S PUFA	44.65±2.22	46.61±3.33 b	46.79±2.51b	40.24±1.50b	45.88±1.40a
S Unsaturated	58.11±1.22	60.43±2.12 b	58.03 ±1.82 a	60.84±2.00 b	60.02±2.21a
18:1/18:0	0.50	0.70	0.61	1.10	0.54
16:1/16:0	0.062	0.047	0.046	0.112	0.080

Values are the mean ± SEM. ANOVA; one-way ANOVA P value:

a: P>0.05, b: P<0.05, c: P<0.01, d: P<0.001 using the post hoc Fischer’s P LSD test

S Saturated: Total saturated fatty acids

S Unsaturated: Total unsaturated fatty acids

S MUFA: Total monounsaturated fatty acids

S PUFA: Total polyunsaturated fatty **acids**

As revealed in Table-3, palmitic acid level (16:0) in the fatty acid composition of the liver increased in D-2, D+AT+AA+LA and AT+AA+LA groups compared to control (*P<*0.001), but stearic acid level decreased in the same groups (*P<*0.001). While the liver palmitoleic acid level (16:1, n-9) did not differ between control, D-1 and D-2 groups, its level increased in the D+AT+AA+LA group (*P<*0.01). Although liver oleic acid level was high (18:1, n-9) in the D+AT+AA+LA and AT+AA+LA groups (*P<*0.01), its level decreased in the D-2 group (*P<*0.05). Liver linoleic acid level (18:2, n-6 LA) was high in the D-1, D- 2 and D+ AT+AA+LA groups (*P<*0.01), however arachidonic acid (20:4, n-6) level was low in the D-2 and D+ D+ AT+AA+LA groups (*P<*0.001). Docosahexaenic acid (22:6, n-3, DHA) level increased in the D-2 and AT+AA+LA groups (respectively, *P<*0.001, *P<*0.05), although there was no differences between control, D-1 and D+AT+AA+LA groups (*P>*0.05) (Table 3).

Table 4 presents changes in muscle fatty acid composition by triple antioxidant combination. The muscle stearic acid level was high in the D-2, D+AT+AA+LA and AT+AA+LA groups (*P<*0.01, *P<*0.001). Muscle oleic acid levels increased in the D-1 group, but its level decreased in the D-2 group (*P<*0.001). While the linoleic acid levels of muscle was high in the D-1 and D-2 groups (*P<*0.01), muscle arachidonic acid level was low in the same groups (*P<*0.05). However, muscle arachidonic acid level increased in the D+AT+AA+LA and AT+AA+LA groups (*P<*0.05). While DHA level in muscle decreased in the D-1 group, its level increased in the D-2 and AT+AA+LA groups (*P<*0.05) (Table 4). 

**Table 4:** The fatty acid composition in muscle tissues of diabetic rats

Fatty acids	Control	D-1	D-2	D+ AT+AA+LA	AT+AA+LA
14:0	0.96±0.15	0.87±0.22	0.86±0.33	0.79±0.20	0.87±0.18
15:0	0.74±0.21	0.34±0.14	0.40±0.12	0.45±0.10	0.90±0.15
16:0	23.46±1.22a	22.01±0.26a	22.48±0.43a	22.57±0.69a	22.95±0.58a
18:0	9.49±0.91a	9.33±0.12a	13.43±1.06d	10.89±0.52b	10.88±1.12c
S Saturated	34.65±1.13a	32.55±1.18b	37.17±0.89b	34.70±1.46a	36.67±1.93b
18:1, n 9	22.40±0.99a	24.12±092c	15.57±1.99d	21.73±1.40 a	20.62±1.49b
18:1, n 7	6.08 ± 0.74	3.71±0.24d	3.61±0.19 d	4.31±0.42c	4.38±0.29 b
S MUFA	28.77±1.80a	28.97±1.20a	19.18±2.00 d	26.04±1.75 b	25.00±1.60b
18:2	21.50±0.74a	27.85±0.93c	28.30±1.11 c	20.42±1.59a	20.24±0.77a
20:4	8.54±026a	7.08±065b	7.00±1.09 b	10.20±0.88 b	9.39±043a
22:6	6.67±0.50	4.77±0.83b	8.41±1.22 b	8.73±0.49 b	9.86±0.93c
S PUFA	36.40±1.50	39.63±2.00b	43.31±2.08 d	39.35±2.48 b	39.49±1.50 b
SUnsaturated	65.38±2.20a	67.46±1.86b	62.89±1.60 b	65.39±1.90a	64.49±1.75b
18:1/18:0	2.95	2.98	1.42	2.39	1.70
S Unsa /S Sat	1.89	2.07	1.66	1.88	1.59

Values are the mean ± SEM. ANOVA; one-way ANOVA P value. a P>0.05, b: P<0.05, c: P<0.01, d: P<0.001 using the post hoc Fischer’s P LSD test.

S Saturated: Total saturated fatty acid

S Unsaturated: Total unsaturated fatty acid

S MUFA: Total monounsaturated fatty acid

S PUFA: Total polyunsaturated fatty acid

## Discussion

Experimental and clinical studies revealed that free radicals are formed disproportionately in diabetes by glucose oxidation, non-enzymatic glycation of proteins (2, 27). Abnormally high levels of free radicals and the simultaneous decline of antioxidant defense mechanisms can lead to damage to cellular organelles and enzymes, increased lipid peroxidation (2), and abnormal lipid, triglycerides and cholesterol levels (28). The polyunsaturated fatty acids in cholesterol esters, phospholipids, and triglycerides are subject to free radical-initiated oxidation and contribute in chain reactions that amplify damage to biomolecules. The present study indicated that cholesterol level in muscle was high in the insulin treated D-1 group. Muscle is a major site of insulin action, and insulin sensitivity may be related to the cholesterol level of muscle lipids. Insulin increases activity of the 3-hydroxy-3-methylglutaryl coenzyme A *reductase *enzyme (HMG-CoA), which is a key enzyme in cholesterol biosynthesis. Therefore, cholesterol synthesis increases and facilitates the storage of fuels and macromolecules in liver, muscle and fat (29). In the present results, the cholesterol level was also found to be high in the insulin and triple antioxidant treated diabetic groups. This augmentation might be a result of insulin administration. However, the level of cholesterol in the liver tissues of AT+AA+LA group was lower than the control group. This decrease may be due to the effects of the administered antioxidants on the cholesterol metabolism. AT could protect polyunsaturated fatty acids, thereby preserving the physical properties of membranes and the environment necessary for enzymatic activities involved in fatty acid and cholesterol metabolism (13). AT supplementation produces a momentous improvement in insulin mediated glucose utilization in healthy people, type-2 diabetics and essential hypertensives (30). In addition, α-tocopherol down-regulates the expression of the cholesterol scavenger receptors (31, 32). 

The results of the present study revealed that the level of GSH in D-2 group was lower than the control group. However, GSH level was high in the treated triple antioxidant group. GSH is the most prevalent low molecular weight antioxidant within cells and it protects cellular constituents from oxidative damage by reacting directly with oxidants or by acting as the substrate for glutathione peroxidase to scavenge peroxides (33). GSH also promotes the antioxidant properties of AA and α -tocopherol (34). When the GSH molecule neutralizes the free radicals, the GSH molecule is converted to oxide form (GSSG). The GSSG is again converted to GSH use to NADPH by the GSH reductase enzyme. The conservation and formation of NADPH in the cells are by the activity of pentose-phosphate pathway and malic enzyme (35). With an insufficient insulin level, the activities of glucose-6-phosphate dehydrogenase in pentose phosphate shunt decrease and glutathione reductase led to impairment of GSH regeneration and increased the level of GSSG. Declined GSH level in the D-2 group may be due to the lack of insulin. Increased GSH level in the triple antioxidant group may be due to the consequences of antioxidants. LA in triple antioxidant mixture has important effects on the glutathione synthesis. Packer *et al* (36) suggested that LA increases the level of glutathione by facilitating the transport of cystine and increases the level of intracellular availability of cysteine. The supply of cysteine is rate limiting for *de novo* synthesis of GSH in many cell types. It has been revealed to be potent antioxidants of the LA, to regenerate through redox cycling other antioxidants like AA and α -tocopherol, and to raise intracellular GSH levels (37, 38). Clinical trials have been shown that LA improved glucose metabolism in diabetic patients and LA treatment also increased insulin sensitivity in type-2 diabetic patients and enhanced glucose transport into skeletal muscle isolated from both obese and lean Zucker rats (18, 39). Yilmaz *et al* (40) found that the level of GSH in erythrocytes of diabetic rats was increased by the combination of the LA, AA 6 palmitate. Dincer *et al* (41) reported that in the LA supplemented group, lipid peroxidation levels were decreased; GSH levels were increased in the liver and pancreas tissues. Powell *et al* (42) suggested that the level of GSH concentrations in the human intestinal epithelial cell line reverted to normal by the administration of LA. Baydas *et al* (43) found that the LA supplementation significantly prevented the increase in lipid peroxidation levels found in diabetic rats, and GSH levels were increased by the administration of LA. Paolisso *et al* (14) suggested that percentage increase in plasma AA levels was correlated with the percentage decline in plasma free radicals and increase in GSH levels. Supplementation with AA, AT and beta-carotene resulted in an improvement of the antioxidative status of kidneys of rats with STZ-induced diabetes (44).

In the FA composition of liver tissue, although palmitic acid levels in D-1 and D-2 and D+AT+AA+LA groups were higher than the control group, stearic acid levels were low in the same groups. In mammalian cells, palmitic and stearic acids are major products of *de novo* synthesis by the activity of cytoplasmic FA synthase (45). The present study showed that the level of 18:1 n-9 in fatty acid composition of liver and muscle tissues was low in the D-2 group. Desaturases are key enzymes in the biosynthesis of the monounsaturated and polyunsaturated fatty acids and thereby contribute to the control of the fatty acid-dependent structure and disorder of the membrane. A relationship has been observed between the 16:0/16:1 and 18:0/18:1 ratios usually used as an index of *in vivo* Δ^9^-desaturase activity. The significant increase in the proportions of 16:1, n-7 and 18:1, n-9 may reflect higher activity of Δ9 desaturase in the cell. An upsurge in the Δ9 desaturase activity has been reported to be related to hyperinsulinemia. It has been found to induce an over-expression of Δ9 desaturase by the effect of insulin in animals (45-47). The level of 18:1 in both liver and muscle tissues of insulin treated diabetic groups was higher than the D-2 group. This increase may be due to the effects of external insulin injection during diabetes duration. Doullet and Ciavatti (4) found that the levels of 16:1 n-9 are induced by diabetes in liver, aorta and plasma. The regulation of Δ9 desaturase is important as physiological and its activity is sensitive to dietary changes, hormonal imbalance etc. (48). 

Lipid peroxidation is a significant aspect of oxidative damage and oxidative modification of phospholipid polyunsaturated fatty acid residues in membrane structure (49). In the current study, while the 18:2, n-6 level in both liver and muscle tissues was high in the D-1, 2 and D+AT+AA+LA groups, arachidonic acid (20:4, n-6) level was low in the D-2 and D+AT+AA+LA groups. However, DHA level was high in the D-2 group. Brenner (50) found the opposite result, and reported that the proportion of this fatty acid increased in all those tissues in insulin-dependent diabetes. In addition, Hu *et al* (51) revealed that the progression of the diabetes mellitus type-1 increased mainly the DHA in the phosphatidylcholine and phosphatidylethanolamine of rat liver tissues. Prolonged diabetes causes profound alterations in the fatty acid composition of phospholipids in liver, heart, kidney, testis, spleen and brain tissues. In poorly controlled diabetes the relative level of 20:4, n-6 decrease and increase in that of LA (18:2). Δ 6/5 desaturases are key enzymes for the synthesis of arachidonic acid and DHA in mammals and they are insulin dependent (6, 50). It has been revealed that the depressed Δ 6 desaturase is restored by insulin stimulation of the gene expression of its mRNA in experimental type-1 diabetes. The depression of Δ 6 and Δ 5 desaturases in diabetes is rapidly correlated by lower contents of arachidonic acid and higher contents of linoleic in almost all the tissues (50). 

Skeletal muscle is a major site of insulin action, and insulin sensitivity may be related to the fatty acid composition of muscle lipids. Mohan *et al* (53) reported that the activities of Δ 6 5 desaturases are depressed in experimental diabetes and in humans with insulin- and non-insulin-dependent diabetes. Doullet and Ciavatti (11) suggested that the α-tocopherol partially corrected Δ^9 ^desaturase activity and consequently enhanced monounsaturated acids in all tissues of experimental diabetes. In the present results, treatment of triple antioxidants to diabetic groups increased the level of monounsaturated fatty acids. This increase might be due to the effects of AT, AA, LA and insulin administration. 

Antioxidant therapy alone had no effect on the measured parameters in either the diabetic or control animals. However, combined insulin and antioxidant therapies normalized blood pressure, plasma MDA and urinary protein in the diabetic animals (25). Insulin therapy alone resulted in incomplete reduction of plasma MDA and blood pressure. Antioxidant therapy was ineffective when given alone, but when combined with insulin treatment, resulted it normalized plasma MDA, blood pressure and reduced urinary protein excretion (54). Although antioxidant treatments can illustrate benefits in animal models of diabetes, evidence from large clinical trials suggests that new and more powerful antioxidants need to be studied to demonstrate whether antioxidants can be effective in treating complications (55). 

## Conclusion

In conclusion, it was observed that GSH levels in liver and muscle tissue of diabetic and non-diabetic rats were increased by triple antioxidant mixture; in particular, the administration of LA increased the level of GSH in non-diabetic rats. In addition, the administration of triple antioxidant mixture and insulin were increased the level of products the SCD in liver tissue.
